# Application of Emerging Teaching Models in Dental Education: A Systematic Review and Meta-Analysis

**DOI:** 10.1016/j.identj.2024.05.016

**Published:** 2024-07-09

**Authors:** Xuefei Pang, Ling Li, Xu Liu, Yan Wang, Bo Yang

**Affiliations:** a Hospital of Stomatology, Guanghua School of Stomatology, Guangdong Provincial Key Laboratory of Stomatology, Sun Yat-sen University, Guangzhou, China; b Guangdong Provincial Key Laboratory of Biomedical Imaging and Guangdong Provincial Engineering Research Center of Molecular Imaging, Department of Infectious Disease, The Fifth Affiliated Hospital, Sun Yat-sen University, Zhuhai, China

**Keywords:** Problem-based learning, Case-based learning, Team-based learning, Flipped classroom, Dental education, Meta-analysis

## Abstract

**Introduction and aims:**

As an experimental teaching method, emerging learning methods including problem-based learning (PBL), case-based learning, team-based learning and flipped classroom (FC) have been widely applied in dental education. This study aims to evaluate the effect of these teaching methods on dental education performance compared to traditional lecture-based learning (LBL).

**Methods:**

The search was carried out in April 2024 in PubMed, EMBASE, Web of Science, and Cochrane Library. All randomized controlled trials were included and the methodological quality assessment was based on the guidelines described in the Cochrane Handbook for Systematic Reviews, followed by a meta-analysis using Stata 14.0 software. Using standard mean deviation (SMD) and 95% confidence interval (95% CI) to determine the effectiveness of emerging teaching methods and LBL in all dental disciplines. Meta-regression was used to analyse sources of heterogeneity. Sensitivity analysis was performed to determine the stability, and Begg's analysis was used to determine whether there is publication bias.

**Results:**

A total of 29 randomized controlled trials including 3502 students were included. The results indicate that emerging educational methods have a significantly positive effect on achieving higher scores (SMD = 0.48, 95% CI = 0.34-0.62, *P* < .001), whether it was theoretical scores (SMD = 0.52, 95% CI = 0.32-0.72, *P* < .001) or skill scores (SMD = 0.45, 95% CI = 0.15-0.76, *P* < .001). Compared to LBL, PBL (SMD = 0.33, 95% CI = 0.01-0.65, *P* = .045) and FC (SMD = 0.50, 95% CI = 0.31-0.69, *P* < .001) can both significantly improve students’ academic performance.

**Conclusion:**

Compared to LBL, emerging educational methods (PBL, case-based learning, and FC) have significantly improved the learning effectiveness of dental students. These emerging educational methods can be advocated and popularized as routine teaching methods.

**Clinical Relevance:**

This study is the first meta-analysis of the effects of emerging teaching methods in dental education which shows great impact of emerging teaching methods on students’ development.

## Introduction

The education in stomatology aims to train stomatologists with rigorous professional knowledge, skilled clinical operational skills, and the ability to critically evaluate their own clinical reasoning and to actively and flexibly learn in clinical work.^1^^,^^2^ However, the current mainstream lecture-based teaching method makes knowledge abstract, which makes it difficult for dental students to grasp knowledge and apply it clinically.^3^ In addition, the lecture mode of teaching is not really conducive to cultivating the innovative thinking and self-directed learning ability of students.^4^

With the rapid development of education, the government attaches more and more attention to the improvement of students’ comprehensive quality. The combination of theoretical knowledge and practical knowledge becomes more and more indispensable for students, especially medical students. Over the past 50 years, many new educational models have emerged and attempted to reconcile teaching with learning, based on the principle that students should lead their own learning process.^5^ Among these new methods, problem-based learning (PBL), team-based learning (TBL), case-based learning (CBL), and flipped classroom (FC) are the most widely used methods, which are similar but have their own characteristics^6^, ^7^, ^8^, ^9^ (Table 1). These methods are designed to improve students’ active learning, collaborative learning, and self-directed learning abilities, so that students can take more responsibility for their own learning and become lifelong learners.^10^^,^^11^ They have been widely incorporated into various medical disciplines with good results, such as surgery, nursing, pharmacy, basic medicine.^12^, ^13^, ^14^, ^15^, ^16^

**Table 1:** The similarities and differences between PBL, CBL, TBL, and FC.

Method	Similarities	Differences
PBL	(1) Student-centred: these three teaching methods emphasize the central position of students and focus on the participation and initiative of students; (2) Group cooperation: these three teaching methods require students to discuss and solve problems in small groups; (3) Situational learning: these three teaching methods emphasize placing learning in specific problems or case situations, enabling students to make connections between what they learn and actual situations.	PBL is problem-oriented and emphasizes students’ active exploration, problem-solving, and critical thinking.
CBL		CBL is case-oriented that trains students to serve clinical practice through the use of real clinical cases, focusing on the combination of students’ knowledge and practice, and improving students’ ability to solve practical problems.
TBL		TBL focuses on teamwork and communication skills.
FC		FC, a more student-centred teaching mode, can motivate positive learning, requiring students to positively participate in the class, engage with learning materials, and cooperate with classmates.

PBL: problem-based learning; CBL: case-based learning; TBL: team-based learning; FC: flipped classroom.

At present, there are more and more research on the effect of these teaching methods in dental education, but the conclusions are not uniform. Many studies believe that PBL and other emerging teaching methods are conducive to the improvement of student achievement,^17^, ^18^, ^19^ but some scholars still point out that there is no significant difference in student achievement in classes with different teaching methods.^20^^,^^21^ Therefore, the actual teaching effect of these new teaching methods in dental education still needs further research.

To systematically ascertain whether students major in dentistry can benefit from these emerging teaching methods, we conducted this systematic review and meta-analysis. The aim of our study was to evaluate the effect of these teaching methods on dental education performance compared to traditional teaching method lecture-based learning (LBL).

## Methods

### Protocol and registration

According to the Meta-analysis of Observational Studies in Epidemiology guidelines^22^ and the Preferred Reporting Items for Systematic Reviews and Meta-Analyses standard,^23^ we conducted this meta-analysis. It doesn't require ethical approval as the article is a systematic review of educational data previously reported articles without patient data. Our research question of this meta-analysis was: Whether PBL, CBL, TBL, and FC are superior to LBL in dental education?

### Eligibility criteria

PICOS model was used to set the inclusion criteria: (1) P (patients/participants): Students majoring in dentistry who are currently undergoing dental education, including specialized students, undergraduate students, graduate students, interns, trainees, doctors and so on; (2) I (intervention/exposure): PBL, CBL, TBL, and FC; (3) C (comparison/control): Traditional educational method LBL; (4) O (outcome): Participants’ abilities after education, including theoretical scores, clinical skills exams, scientific research abilities, etc.; (5) S (study design): Randomized controlled trial (RCT).

The inclusion criteria are as follows: (1) The research participants must be students majoring in dentistry, and the subjects of study must be content that students in that major must master during their study period; (2) The study design is RCT; (3) The research objective is to compare the theoretical teaching effectiveness and practical teaching effectiveness of learning courses (effectiveness is measured by exam scores in this study; practical teaching is an educational strategy that aims to deepen students’ understanding and mastery of knowledge through practical operation); (4) The experimental group is an emerging educational tool that only includes PBL, CBL, TBL and FC without other educational tools, but allows for auxiliary tools such as social media; the control group used the traditional teaching method based on lecture, which we defined as LBL and also allowed for the assistance of auxiliary tools. (5) The research results need to include student abilities presented in data format, with data type being mean ± standard deviation (SD) or other data that can be converted into mean ± SD format. Studies that do not have readily available raw data, but could be calculated, are also included.

Any studies that do not meet the inclusion criteria mentioned above were excluded. Two reviewers independently evaluated to screen potential studies. If there was a dispute, the third reviewer would discuss and resolve the differences until a consensus is reached.

### Information sources and search strategy

Following the search strategy shown in Supplemental Table 1, we conducted a comprehensive and detailed search on four databases (PubMed, EMBASE, Web of Science, and Cochrane Library, from their inceptions until April 20, 2024) with the aim of exploring the differences in teaching effectiveness between PBL, CBL, TBL, and FC compared to LBL, without any restrictions on article language or research region.

### Study selection and data collection

The research data was collected and extracted using predefined data table (Supplemental Table 2) by two independent reviewers and then checked. If there was any inconsistency in data processing, it was negotiated until a consensus was reached. In addition, the following information from studies that meet the standards were also extracted and summarized: (1) the first author; (2) year of publication; (3) study type; (4) study participants; (5) sample size (experimental group and control group); (6) the teaching methods adopted by the experimental group; (7) the teaching methods adopted by the control group; (8) learning curricula/content/skills; (9) capability evaluation indicators (exam scores); (10) number of problems/cases; (11) credit hour in experimental group and control group; (12) auxiliary teaching tools or materials; (13) learning objectives. During the process of extracting the above information, any unresolved conflicts between the two reviewers were resolved through the intervention of a third reviewer.

### Risk of bias and applicability assessment

Outcome indicators, namely the extracted mean ± SD scores of the study population after learning, were compiled together with the data from subgroup analysis in duplicate. According to Cochrane Handbook for Systematic Reviews (http://www.cochranelibrary.com/), two researchers independently evaluated the methodological quality and bias risk of the included study, providing reference for the quality grading of evidence. These parameters were considered in this specific study as the meaningful evaluation index: (1) random sequence generation; (2) allocation concealment; (3) blinding; (4) incomplete outcome data; (5) selective reporting; (6) other sources of bias contained. The results of each evaluation were divided into ‘low bias risk’, ‘high bias risk’, and ‘uncertain bias risk’. All of the included articles have considered the risk factors. All necessary information was extracted from the published content of the article, including Supplemental Materials. If there was additional information that needs to be obtained, we would try to contact the author for assistance. When the opinions of the two reviewers were not consistent, discuss first. If the conflict could not be resolved, the third reviewer would participate (Table 2).

**Table 2:** Risk of bias assessment.

Source	Adequate sequence generation	Allocation concealment	Blinding	Incomplete outcome data addressed	Free of selective reporting	Free of other bias	Description of other bias
Moreno-Lopez et al.^25^	Yes	Unclear	No	Yes	Yes	No	Teacher's identity; transfer of knowledge between groups
Callis et al.^26^	Unclear	Unclear	No	Yes	Yes	No	Different courses; different participation times
Zhang et al.^27^	Unclear	Unclear	No	Yes	Yes	Yes	-
Liu et al.^28^	Yes	Unclear	No	Yes	Yes	Yes	-
Ilguy et al.^29^	No	Unclear	No	Yes	Yes	Yes	-
Wu et al.^30^	Yes	Unclear	No	Yes	Yes	Yes	-
Su et al.^31^	No	Unclear	No	Yes	Yes	Yes	-
Chutinan et al.^32^	Unclear	Unclear	No	Yes	Yes	Yes	-
Sun et al.^33^	Yes	Unclear	No	Yes	Yes	Yes	-
Montero et al.^34^	No	Unclear	No	Yes	Yes	Yes	-
Zhu et al.^35^	No	Unclear	No	Yes	Yes	Yes	-
Xiao et al.^36^	Unclear	Unclear	No	Yes	Yes	Yes	-
Fleagle et al.^37^	Unclear	Unclear	No	Yes	Yes	Yes	-
Isherwood et al.^38^	Yes	Yes	No	Yes	Yes	Yes	-
Siegel et al.^39^	Yes	Unclear	No	Yes	Yes	Yes	-
Zhu et al.^40^	Unclear	Unclear	No	Yes	Yes	Yes	-
Qutieshat et al.^41^	Unclear	Unclear	No	Yes	Yes	Yes	-
Li et al.^42^	No	Unclear	No	Yes	Yes	Yes	-
Rocha et al.^43^	Yes	Unclear	No	Yes	Yes	Yes	-
Gallardo et al.^44^	Yes	Unclear	No	Yes	Yes	Yes	-
Sivarajan et al.^45^	Yes	Yes	No	Yes	Yes	Yes	-
Lau et al.^46^	No	Unclear	Yes	Yes	Yes	Yes	-
Li et al.^47^	Unclear	Unclear	No	Yes	Yes	Yes	-
Zhou et al.^48^	Yes	Unclear	Yes	Yes	Yes	Yes	-
Zhong et al.^17^	Unclear	Unclear	No	Yes	Yes	Yes	-
Luo et al.^49^	Yes	Unclear	No	Yes	Yes	Yes	-
Karaca et al.^18^	No	Unclear	No	Yes	Yes	Yes	-
Inamochi et al.^19^	Yes	Unclear	Yes	Yes	Yes	Yes	-
Li et al.^50^	No	Unclear	No	Yes	Yes	Yes	-

### Data synthesis

Analyse the extracted data using Stata 14.0 software. *P* values were two-sided, and the significance level was set at 0.05. Due to the extraction of continuous results from different scales and the fact that the full marks included in the study were not always 100 points, we chose to use standard mean deviation (SMD) and corresponding 95% confidence intervals (95% CIs) for evaluation to generate forest plots and display the results. Due to the heterogeneity among the included researchers, a random effects model was chosen. Taking 50% as the boundary, less than indicates less heterogeneity, while the opposite indicates substantial heterogeneity.^24^ Evaluate the impact of different educational methods, grades, teaching subjects, and assessment content on the results through subgroup analysis, and analyse potential heterogeneity. Further analyse the sources of heterogeneity through meta-regression. Use sensitivity analysis to test the stability of the study and observe its impact on overall results by sequentially deleting each study. Begg's funnel plot is used to detect publication bias, and when the *P* value is less than 0.05, it indicates a significant publication bias in the included study.

## Results

### Literature search and study characteristics

**Fig. 1:**
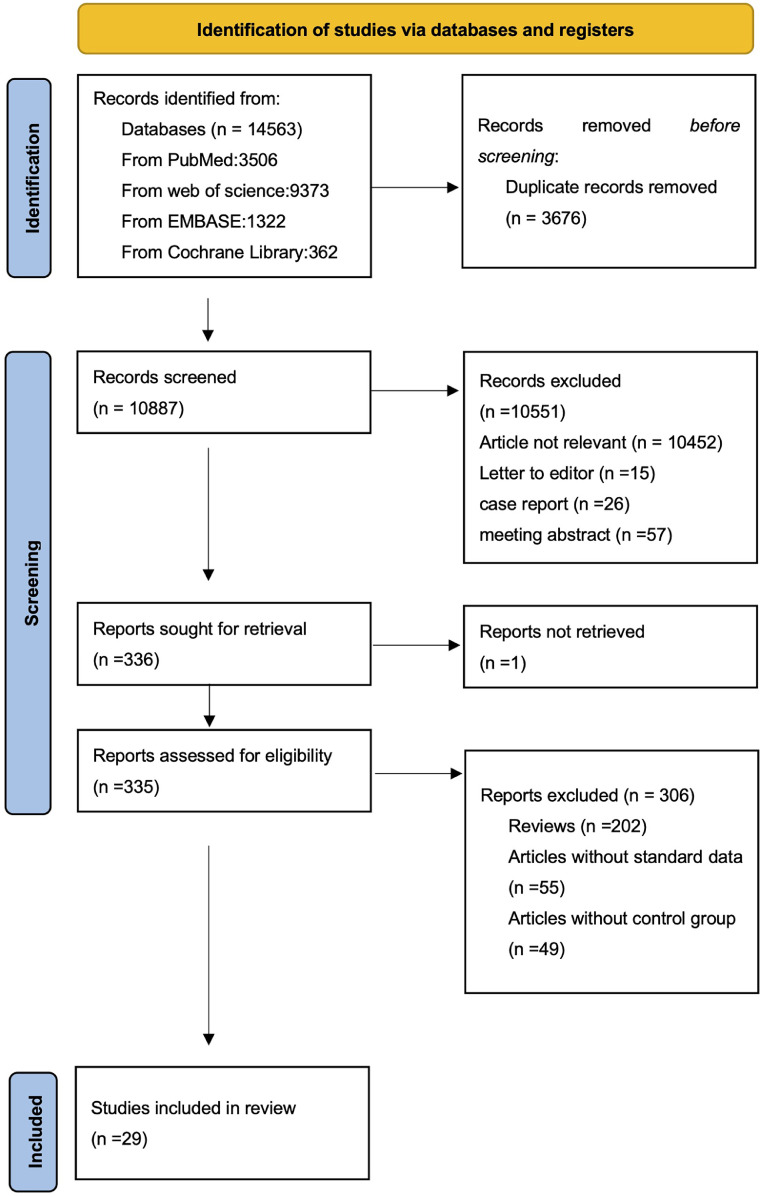
Flow diagram of studies included in the systematic review and meta-analysis.

After searching four databases, a total of 14,563 articles were needed to be screened, and after excluding duplicate articles, there were still 10,887 articles to be reviewed. We read the titles and abstracts of all articles and found that there are 335 articles which needed further evaluation. Through reading the full text, 55 studies lacked the data we requested, 49 studies did not have a control group or their control group did not use LBL as the learning method, 202 articles were reviews. Ultimately, 29 studies were included in our meta-analysis with a total of 3502 participants (Figure 1).

The specific characteristics of the 29 articles are shown in Table 3 and Supplemental Table 3. They are all RCT studies from different countries and they presented both experimental learning method groups (PBL, CBL, and FC) and control group (LBL). Among the 3502 participants, 1696 students studied used new teaching methods (PBL in 6 studies, CBL in 4 studies, FC in 16 studies, combined study methods in 3 studies), and 1806 students were assigned to the LBL group. According to our search strategy and inclusion criteria, no articles only using TBL for the experimental group teaching methods were included. The research population includes dental undergraduates in every grade (including interns), residents, and doctors. Among them, 7 studies were conducted on the 3rd-year undergraduates, 7 studies were conducted on the 4th-year undergraduates, 5 studies were conducted on the 5th-year undergraduates and 3 studies were conducted on the doctors. Apart from that, there was 1 study conducted on 1st-year and 2nd-year undergraduates, respectively. The study subjects included prosthodontics (6 studies) orthodontics (4 studies), endodontics (4 studies), oral and maxillofacial surgery (2 studies), etc. The assessment of students’ final abilities included exams for theoretical knowledge, clinical skills, essay writing, and so on. After quality assessment, we believed that there are no articles of very low quality, so we have not excluded them.

**Table 3:** Characteristics of studies included in the analysis.

Study	Year	Study type	Country	Participants	Sample size (study)/(control)	Experimental group	Control group	Curricula	Outcome indicator
Moreno-Lopez et al.^25^	2009	RCT	Spain	5th-y students	15/36	PBL	LBL	Special Care in Dentistry	Examination; time devoted
Callis et al.^26^	2010	RCT	America	3th-y students	31/40	Hybrid-PBL	LBL	Physiology, neurology, pharmacology, and biochemistry	Essay examination
Zhang et al.^27^	2012	RCT	China	4th-y students	43/44	PBL	LBL	Oral and maxillofacial surgery	Clinical Skill Test
Liu et al.^28^	2013	RCT	China	5th-y students	20/21	CBL	LBL	Endodontics	Examination
Ilguy et al.^29^	2014	RCT	Turkey	4th-y students	54/55	CBL	LBL	Oral diseases course	Case examination
Wu et al.^30^	2014	RCT	China	undergraduates	41/41	CBL and PBL	LBL	Dentistry	Examination
Su et al.^31^	2017	RCT	China	4th-y students	22/21	PBL	LBL	Orthodontics	-
Chutinan et al.^32^	2018	RCT	America	2nd-y doctors	70/70	FC	LBL	Dental anatomy	Tooth waxing, tooth identification assessments, and written examination scores
Sun et al.^33^	2018	RCT	China	residents	26/25	FC-CBL	LBL	Endodontics	Examination
Montero et al.^34^	2018	RCT	Spain	4th-y students	57/46	Hybrid-PBL	LBL	Prosthodontics	academic performance
Zhu et al.^35^	2018	RCT	China	4th- and 5th-y students	46/45	FC	LBL	Cardiopulmonary resuscitation	Didactic test and skill evaluation
Xiao et al.^36^	2018	RCT	America	1st-y Doctors	141/142	FC	LBL	Physiology	Identical quiz
Fleagle et al.^37^	2018	RCT	America	1st-y doctors	241/242	FC	LBL	Human gross anatomy	Laboratory examinations
Isherwood et al.^38^	2019	RCT	Britain	5th-y students	31/30	FC	LBL	Orthodontics	Examination
Siegel et al.^39^	2019	RCT	America	3th-y students	32/32	FC	LBL	Prosthodontics	Crown preparations and only preparations
Zhu et al.^40^	2020	RCT	China	3th-y students	32/31	FC	LBL	Ophthalmology	Skill exams
Qutieshat et al.^41^	2020	RCT	Jordan	4th-y students	253/364	FC	LBL	Conservative dentistry and clinical dental skills	Test scores
Li et al.^42^	2021	RCT	China	1st-y students	106/106	CBL	LBL	Cariology	Cavity RCT Preparation Skill Evaluation
Rocha et al.^43^	2021	RCT	Brazil	2nd-y students	16/16	PBL-Social media	LBL	Radiographic diagnosis of proximal carious lesions	Postmethodology test
Gallardo et al.^44^	2021	RCT	Spain	Dental students	39/37	FC	LBL	Paediatric dentistry	Questionnaire
Sivarajan et al.^45^	2021	RCT	Malaysia	3th-y students	20/20	FC	LBL	Orthodontics	Orthodontic wire-bending scores
Lau et al.^46^	2021	RCT	Malaysia	3th-y students	20/20	FC	LBL	Orthodontics	Adams clasp wire-bending exercise scores
Li et al.^47^	2022	RCT	China	5th-y students	22/28	CBL-Rain Classroom	LBL	Prosthodontics	Examination
Zhou et al.^48^	2022	RCT	China	4th-y students	30/30	WeChat-based FC	LBL	Endodontics	Root canal filling and on-site quiz
Zhong et al.^17^	2023	RCT	China	3th-y students	110/104	FC	LBL	Oral histopathology	Written theory tests; oral histopathology slide tests
Luo et al.^49^	2023	RCT	China	5th-y students	44/43	FC	LBL	Prosthodontics	Theoretical achievements; Practical achievements
Karaca et al.^18^	2023	RCT	Turkey	3th-y students	44/39	FC (remotely and online)	Virtual LBL (remotely and online)	Pedodontics	Academic achievement test
Inamochi et al.^19^	2023	RCT	Japan	4th-y students	70/67	FC	LBL	Prosthodontics	Individual and group tests
Li et al.^50^	2024	RCT	China	Oral and maxillofacial oncology students	20/20	PRI-E	LBL	Oral and maxillofacial oncology	Final test scores; questionnaire assessments

RCT: randomized controlled trial; PBL: problem-based learning; LBL: lecture-based learning; CBL: case-based learning; FC: flipped classroom.

### Effects of PBL/CBL/FC on examination scores

**Fig. 2:**
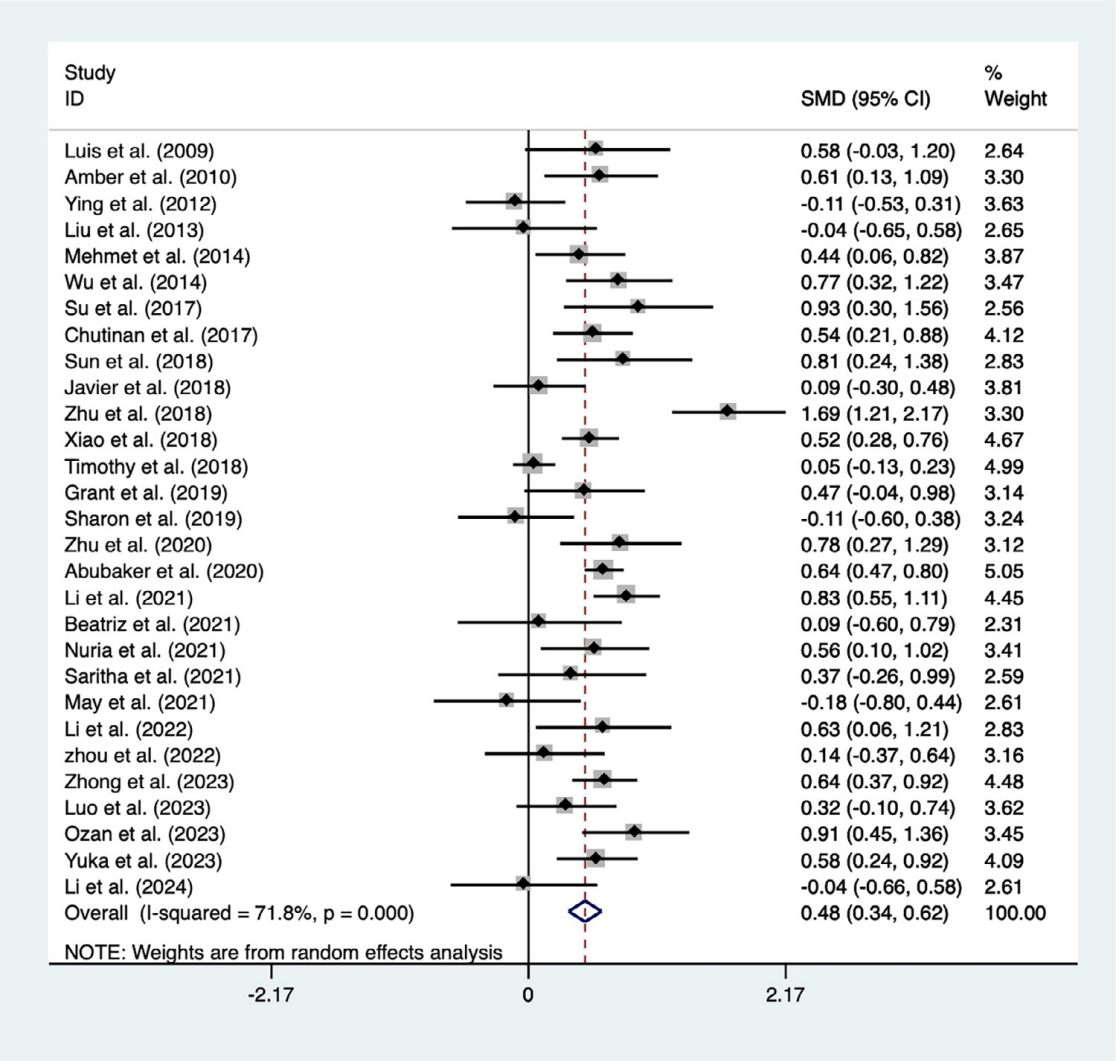
Forest plot for the meta-analysis of outcome indicator. In the forest plot, the black square represents the point estimate of the SMD value of the study, the size of the square represents the weight of the study, the horizontal line represents the 95% confidence interval of the SMD value of the study, and the diamond represents the combined effect value of the study.

**Fig. 3:**
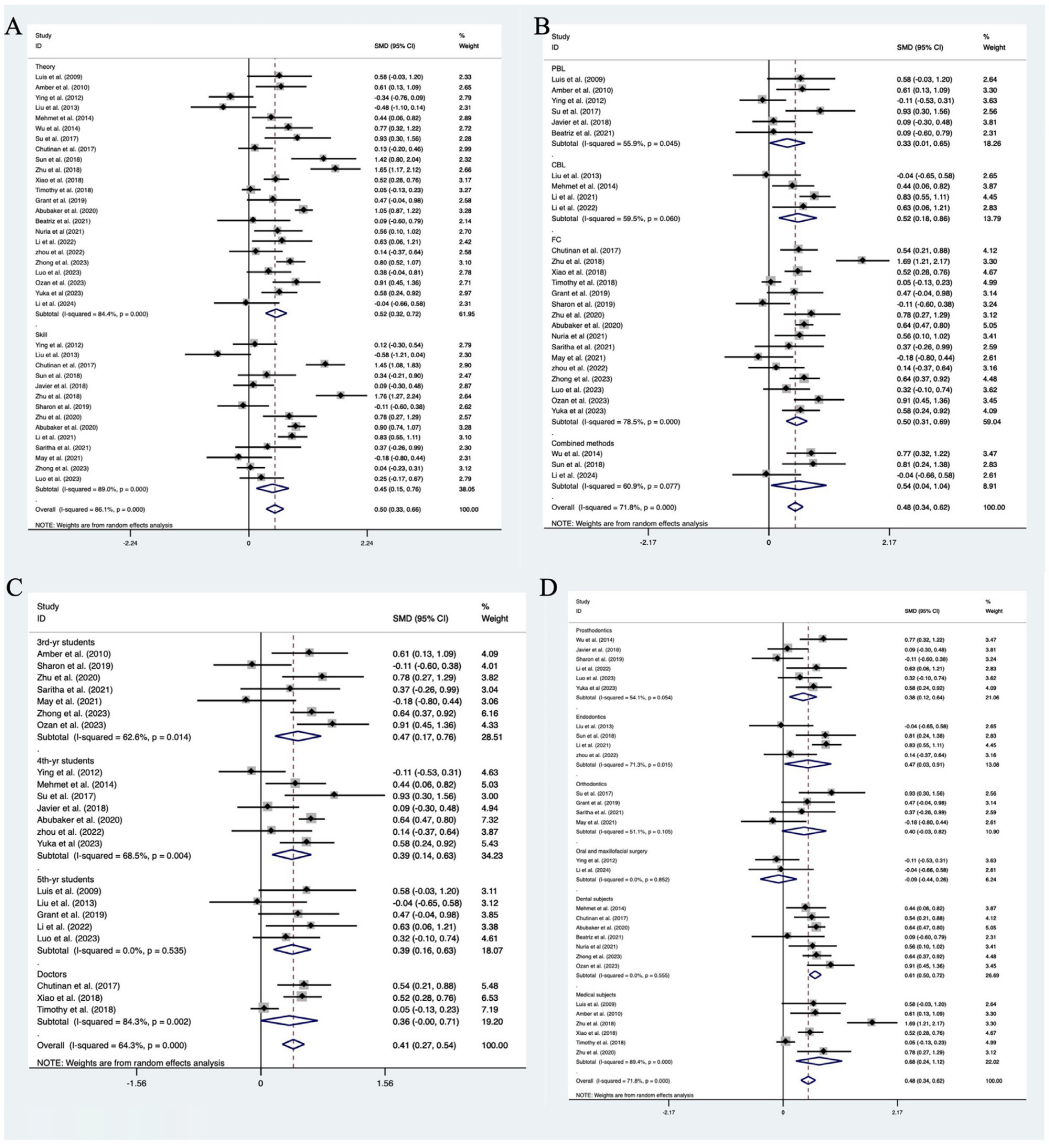
Forest plot of subgroup analysis showing the effect of different assessment content (theory or skills) (A), study methods (B), students’ grades (C), and different subjects (D) on dental education.

A meta-analysis incorporating 29 studies showed that compared to traditional teaching method LBL, emerging teaching methods can significantly improve students’ exam scores (SMD = 0.48, 95% CI = 0.34-0.62, *I*^2^ = 66.4%, *P* < .001) (Figure 2), whether it was theoretical scores (SMD = 0.52, 95% CI = 0.32-0.72, *P* < .001) or clinical skill scores (SMD = 0.45, 95% CI = 0.15-0.76, *P* < .001) (Figure 3A).

Based on different teaching methods, we conducted subgroup analysis and the results showed that both PBL (SMD = 0.33, 95% CI = 0.01-0.65, *P* = .045) and FC (SMD = 0.50, 95% CI = 0.31-0.69, *P* < .001) had significantly higher teaching effects than traditional teaching method LBL. CBL (SMD = 0.52, 95% CI = 0.18-0.86, *P* = .060) and combined methods (using more than 2 kinds of emerging teaching methods) (SMD = 0.54, 95% CI = 0.04-1.04, *P* = .077) are also superior to LBL, but the results are not significant (Figure 3B).

Based on different types of students, we conducted subgroup analysis. We found that 3rd-year undergraduates (SMD = 0.47, 95% CI = 0.17-0.76, *P* = .014), 4th-year undergraduates (SMD = 0.39, 95% CI = 0.14-0.63, *P* = .004) and doctoral students (SMD = 0.36, 95% CI = 0.000-0.71, *P* = .002) can all achieve significant improvement in grades. However, the improvement in grades of 5th-year undergraduate students (SMD = 0.39, 95% CI = 0.16-0.63, *P* = .535) is not significant (Figure 3C).

When conducting subgroup analysis with the subject of study as the variable, although students were able to improve their grades in prosthodontics (SMD = 0.38, 95% CI = 0.12-0.64, *P* = .054), endodontics (SMD = 0.47, 95% CI = 0.03-0.91, *P* = .015), other dental subjects (SMD = 0.61, 95% CI = 0.50-0.72, *P* = .555), and nondental medical subjects (SMD = 0.68, 95% CI = 0.24-1.12, *P* < .001), significance only existed in endodontics and nondental medical subjects learning. However, there was no difference between emerging teaching methods and LBL in the study of orthodontics (SMD = 0.40, 95% CI = −0.03 to 0.82, *P* = .105) and oral and maxillofacial surgery (SMD = −0.09, 95% CI = −0.44 to 0.26, *P* = .852) (Figure 3D).

### Meta-regression

Potential sources of heterogeneity between studies were detected by meta-regression. The results showed that none of the learning methods (*P* = .458) of the experimental group, the students’ grades (*P* = .861), and the subjects of study (*P* = .337) were the sources of heterogeneity in the results. We need more research to analyse heterogeneity.

### Sensitivity analysis and publication bias

**Fig. 4:**
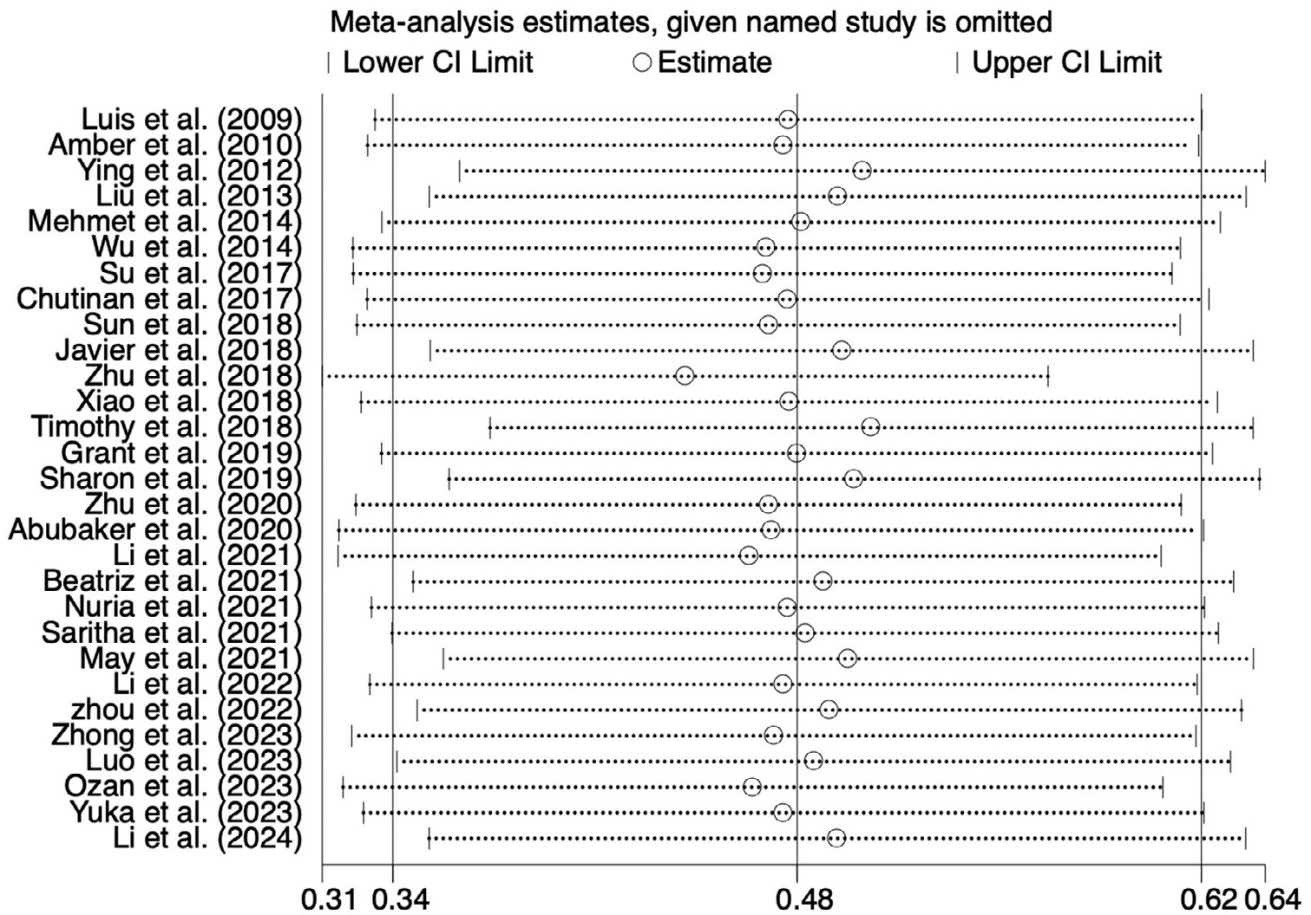
Sensitivity analysis. The pooled SMDs and 95% CIs were stable after the deletion of each study in the analysis.

**Fig. 5:**
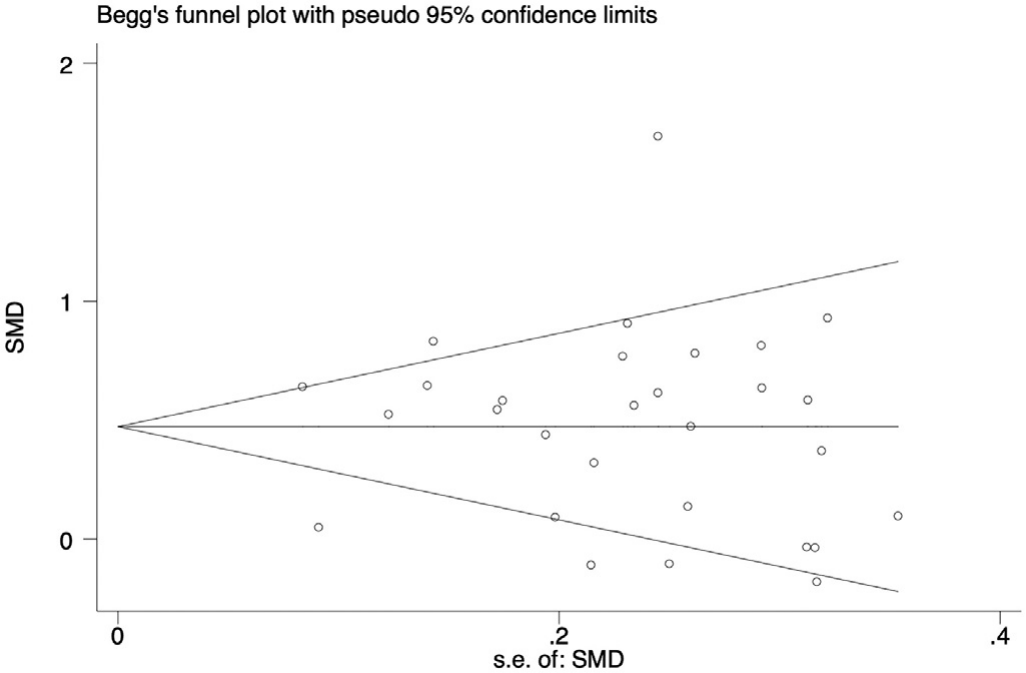
Begg's funnel plot analysis to detect publication bias.

Due to the high risk of bias in some included articles, we used sensitivity analysis to evaluate the stability of the meta-analysis results. We tested the stability of our findings by evaluating the statistical results after sequentially deleting each study. The sensitivity analysis results showed no significant change in the overall results of heterogeneity, indicating that our results were statistically reliable (Figure 4). The Begg's test of SMD relative to SMD standard error did not show evidence of publication bias in exam scores (*P* = .302) (Figure 5). The results of the Egger test were similar (*P* = . 926).

## Discussion

The emerging teaching methods are effective teaching tools implemented in various subjects, dentistry, health care education, nursing, medical radiation science education, etc. Different from traditional teaching methods, the emerging teaching methods are student-centred and effective in promoting students’ critical thinking skills, problem-solving skills, operation skills, and learning skills, and converting passive learning into active learning. The emerging teaching methods have a bright future.

### General interpretation of main results

In this meta-analysis, a total of 29 RCTs were included. The key findings of this meta-analysis suggested that emerging teaching methods such as PBL, CBL, and FC, had a more positive effect on students’ exam scores compared to LBL in dental education. Besides, most of the articles included indicated that students educated in curricula that use emerging teaching methods mastered knowledge better than those who educated in curricula that use traditional teaching methods. In the present study, the emerging teaching methods proved to be useful tools for improving the competence of dental education students. The advantages of these teaching methods were obvious, but their disadvantages could not be ignored, which can provide a direction for further improvement.

### Strengths and limitations of the meta-analysis

To our knowledge, this study is the first systematic review and meta-analysis of the effects of emerging teaching methods versus traditional teaching methods in dental education. This study integrated many different countries’ articles for analysis and came to a more convincing conclusion that new educational methods had significantly improved the academic performance of dental students compared to LBL. With the emergence of new educational models, the shortcomings of traditional teaching models are gradually exposed. Traditional dental educational methods are mostly teacher-centred, allowing students to learn as much as possible, which makes students lack the ability to take the initiative to learn and think independently by themselves. In contrast, the emerging educational models are student-centred and can motivate students to actively learn and acquire information, find effective learning skills to achieve study goals, and enhance exam scores, which is demonstrated by the results of this article. The present study showed that dental students using emerging teaching methods got better exam scores than those participated in LBL. Therefore, the emergence of new educational models might provide a new direction for China's educational reform, which could help students cultivate better professional skills.

There are some limitations of the present study. First, lack of large-scale sample sizes in the included studies on the effects of emerging teaching methods in dental education is an obvious limitation. The small sample size of the experimental group or control group would affect the accuracy and validity of the analysis. Second, the heterogeneity of this study is *I*^2^ = 66.4%, indicating a high level of heterogeneity. However, the use of meta-regression analysis did not identify the source of heterogeneity among existing covariates, and more research is needed to be included in the analysis. Third, some of the included studies were not strictly randomized, and because of the particularity of the experiment, it was impossible for researchers to implement blinding method and allocation concealment through the whole experiment. Therefore, the selection bias and performance bias were unavoidable.

### Discuss implications of the results for practice, policy, and future research

In light of the study, we know that the emerging teaching methods have better performance than traditional teaching model. This suggests that we can further promote the application of these emerging teaching methods in Chinese dental education by reforming the educational system. However, the emerging teaching methods are not without weaknesses. The emerging teaching methods sometimes are difficult to put into effect because they require a large amount of money and time, and hard to see results in a short time. The emerging teaching methods should be improved constantly to make up for their strengths and weaknesses in order to facilitate the development of education. Some experts combined emerging educational models with traditional teaching models to expand their strengths and weaken their weaknesses. This might a better teaching method in the future.

## Author contributions

Xuefei Pang and Ling Li contributed to conception, design, and data acquisition and interpretation; drafted and critically revised the manuscript. Additionally, Yan Wang, Xu Liu, and Bo Yang provided suggestions for revision and contributed to design and interpretation.

## Conflict of interest

The authors declare that they have no known competing financial interests or personal relationships that could have appeared to influence the work reported in this article.

## Appendix Supplementary materials

Image, application 1

Image, application 2

Image, application 3

## Footnotes

Supplementary material associated with this article can be found in the online version at doi:10.1016/j.identj.2024.05.016.
