# Supplementary material for: Application of Emerging Teaching Models in Dental Education: A Systematic Review and Meta-Analysis
Source: Int Dent J. 2024 Jul 9;74(6):1185–96. doi: 10.1016/j.identj.2024.05.016 (PMC11551604; doi:10.1016/j.identj.2024.05.016)
Supplement: Supplementary file 1 [file mmc1.docx]

Supplementary Table 1 Search strategies for databases.

| Database | Date of coverage | Search strategy |
| --- | --- | --- |
| Pubmed  3506 | until April 20， 2024 | (dental OR dentist OR dentists OR dentistry OR oral OR stomatology OR endodontics OR periodontology OR (pediatric stomatology) OR (oral preventive medicine) OR (oral mucosal disease) OR (oral and maxillofacial surgery) OR orthodontics OR prosthodontics OR (oral implantology)) AND (PBL OR (problem based learning) OR (problem-based learning) OR CBL OR (case based learning) OR (case-based learning) OR TBL OR (team based learning) OR (team-based learning) OR (flipped classroom)) |
| Web of Science  9373 | until April 20， 2024 | #1 TS=(dental) OR TS=(dentist) OR TS=(dentists) OR TS=(dentistry) OR TS=(oral) OR TS=(stomatology) OR TS=(endodontics) OR TS=(periodontology) OR TS=(pediatric stomatology) OR TS=(oral preventive medicine) OR TS=(oral mucosal disease) OR TS=(oral and maxillofacial surgery) OR TS=( orthodontics) OR TS=(prosthodontics) OR TS=(oral implantology)  #2 TS= (PBL) OR TS=(problem based learning) OR TS=(problem-based learning) OR TS=(CBL) OR TS=(case based learning) OR TS=(case-based learning) OR TS=(TBL) OR TS=(team based learning) OR TS=(team-based learning) OR TS=(flipped classroom)  #3 #1 AND #2 |
| EMBASE  1322 | until April 20， 2024 | #1 dental:ti,ab,kw OR dentist:ti,ab,kw OR dentists:ti,ab,kw OR oral:ti,ab,kw OR stomology:ti,ab,kw OR endodontics:ti,ab,kw OR periodontology:ti,ab,kw OR 'pediatric stomatology':ti,ab,kw OR 'oral preventive medicine':ti,ab,kw OR 'oral mucosal disease':ti,ab,kw OR 'oral and maxillofacial surgery':ti,ab,kw OR orthodontics:ti,ab,kw OR prosthodontics:ti,ab,kw OR 'oral implantology':ti,ab,kw  #2 PBL:ti,ab,kw OR 'problem based learning':ti,ab,kw OR 'problem-based learning':ti,ab,kw OR CBL:ti,ab,kw OR 'case based learning':ti,ab,kw OR 'case-based learning':ti,ab,kw OR TBL:ti,ab,kw OR 'team based learning':ti,ab,kw OR 'team-based learning':ti,ab,kw OR 'flipped classroom':ti,ab,kw  #3 #1 AND #2 |
| Cochrane Library  362 | until April 20， 2024 | #1 (dental):ti,ab,kw OR (dentist):ti,ab,kw OR (dentists):ti,ab,kw OR (oral):ti,ab,kw OR (stomology):ti,ab,kw OR (endodontics):ti,ab,kw OR (periodontology):ti,ab,kw OR (pediatric stomatology):ti,ab,kw OR (oral preventive medicine):ti,ab,kw OR (oral mucosal disease):ti,ab,kw OR (oral and maxillofacial surgery):ti,ab,kw OR (orthodontics):ti,ab,kw OR (prosthodontics):ti,ab,kw OR (oral implantology):ti,ab,kw  #2 (PBL):ti,ab,kw OR (problem based learning):ti,ab,kw OR (problem-based learning):ti,ab,kw OR (CBL):ti,ab,kw OR (case based learning):ti,ab,kw OR (case-based learning):ti,ab,kw OR (TBL):ti,ab,kw OR (team based learning):ti,ab,kw OR (team-based learning):ti,ab,kw OR (flipped classroom):ti,ab,kw  #3 #1 AND #2 |
