# Supplementary material for: Application of Emerging Teaching Models in Dental Education: A Systematic Review and Meta-Analysis
Source: Int Dent J. 2024 Jul 9;74(6):1185–96. doi: 10.1016/j.identj.2024.05.016 (PMC11551604; doi:10.1016/j.identj.2024.05.016)
Supplement: Supplementary file 3 [file mmc3.docx]

Supplementary Table 3 Characteristics of studies included in the analysis.

| Study | Number of problems/cases | Credit hour in experimental group | Credit hour in control group | other pedagogies | other support learning materials | learning objectives |
| --- | --- | --- | --- | --- | --- | --- |
| Luis et al.(25) | One problem | 1 hour per week for 10 weeks | 1 hour per week for 10 weeks | Experimental group studied in 3 groups of 5 | None | Learn special care in dentistry |
| Amber et al.(26) | Unknow | Unclear times for 5 months | Unclear times for 5 months | None | None | Assessed the ability of applying the basic science knowledge acquired to clinical scenarios |
| Ying et al.(27) | Unknow | 24 hours in 1 year | 24 hours in 1 year | Supervised 1-year clinical rotation in both groups; Experimental group studied in 5 groups | None | Teach oral and maxillofacial surgery knowledge and surgical skills |
| Liu et al.(28) | Three cases | 3 times in 11 months | 30～40 minutes per time for 6 times in 11 months | Supervised 11-months clinical rotation in both groups | None | Improve students' clinical operation ability in endodontics |
| Mehmet et al.(29) | Unknow | Unclear times in 1 year | Unclear times in 1 year | Experimental group studied in group of 8 | None | Improve students’ clinical decision making ability |
| Wu et al.(30) | Unknow | 3 hours per times for 10 times in 11 months | Unclear times in 11 months | Experimental group studied in group of 3～4 | None |  |
| Su et al.(31) | One problem | 3 h | 3 h | Both groups studied in 5 groups of 4～5 | Multimedia, pictures, audio and video | Improve the ability of solving the clinical problems of orthodontics |
| Chutinan et al.(32) | / | 5 times | 5 times | Experimental group studied in group | Videos, Learning Catalytics | Recognise the characteristics of tooth morphology, identify natural dentition and use their knowledge of dental anatomy to create tooth morphology in waxing exercises |
| Sun et al.(33) | Unknow | Unknow | Unknow | Experimental group studied in 4 groups of 6～7 | Textbook, literature, video, audio; Wechat | Cultivate the ability of endodontics |
| Javier et al.(34) | Unknow | 2 hours per time per week when learning theory; 3 hours per time per week when learning preclinical and clinical skills | 2 hours per time per week when learning theory; 3 hours per time per week when learning preclinical and clinical skills | Both groups studied in group of 3 when learning preclinical and clinical skills | Video, seminar | Gain competence in prosthodontics |
| Zhu et al.(35) | / | 8 credit hours | 8 credit hours | Micro-class, both groups studied in group | Videos, wechat, Sim Man3G | Master Cardiopulmonary resuscitation skills |
| Xiao et al.(36) | / | 2.5 hours | 2.5 hours | Experimental group studied alone or in group | VoiceThread, online module, content-based questions, cases, | Study the autonomic nervous system (ANS) in a physiology course |
| Timothy et al.(37) | / | 125 hours in one semester | 125 hours in one semester | Both groups studied in group | Videos, Panopto Video Platform server | Learn to be effective in the gross anatomy laboratory |
| Grant et al.(38) | / | 6 videos | 60 mins | None | Videos, study material, | Train the ability to manage orthodontic emergencies |
| Sharon et al.(39) | / | 1249 mins | 1169 mins | Experimental group included conveying a two-hour time-in-chair appointment target | Faculty Assessment Form, | Reduce the students’ time to complete CAD/CAM indirect restorations without compromise the desired quality of the CAD/CAM tooth preparations |
| Zhu et al.(40) | / | 45 mins per times for 5 times per weeks in 6 weeks | 45 mins per times for 5 times per weeks in 6 weeks | Experimental group studied in group, PBL and LBL | Videos, case discussion | Learn about ophthalmology |
| Abubaker et al.(41) | / | 7 credit hours | 7 credit hours | Experimental group studied in patient role-playing, audience response system questions and interactive sessions | Weekly material, e-lectures, screencasts, course's forum | Possess the clinical skills necessary to practice dentistry, be proficient in critically evaluating their own clinical reasoning and understand the rationale behind each dental procedure |
| Li et al.(42) | More than 20 cases | One semester | One semester | Students were divided into several small WeChat groups | Social media (mainly WeChat), pictures, short videos, text descriptions | Dental undergraduate cariology education |
| Beatriz et al.(43) | One problem | 50 mins per times for 2 times | 50 mins per times for 1 time | None | Clinical case, images of dental radiographic examinations | Improve the diagnostic accuracy of dental students in the radiographic detection of proximal carious lesions |
| Nuria et al.(44) | / | A lesson | A lesson | None | Videos, UCM virtual campus (Moodle 3.4.) | Improve understanding and learning of the treatments of paediatric dentistry substantially |
| Saritha et al.(45) | / | 1 time per weeks in 18 weeks | 1 time per weeks in 18 weeks | Both groups studied in group | Videos | Train students’ manual dexterity |
| May et al.(46) | / | 2 hours for 1 lesson in 6 weeks | 2 hours for 1 lesson in 6 weeks | Both groups studied in group | Videos, teaching material posted online, Sudent Powered e-Collaboration Transforming UM（SPECTRUM） | Teach orthodontic wire-bending skills |
| Li et al.(47) | One case | A lesson | A lesson | Rain Classroom; Experimental group studied in group | Mind map | Improve interns’ clinical skills related to complete dentures |
| Zhou et al.(48) | / | A 90mins lesson | A 90mins lesson | Experimental group studied in 5 groups of 6 | Videos, questions | Increase number of well-trained dentists to perform root canal treatment |
| Zhong et al.(17) | / | A lesson | A lesson | Experimental group used Superstar Platform and Virtual Simulation Experiment Teaching Center for Dentistry; Control group studied in group and used Superstar Platform | Videos, face-to-face live demonstration | provide students with an understanding of the nature of diseases |
| Luo et al.(49) | / | A lesson | A lesson | Experimental group studied in group | Videos | Better understand the 3D structure of RPDs and establish clear design ideas |
| Ozan et al.(18) | / | 4 weeks | 4 weeks | None | Videos, case questions, EdPuzzle application, an LMS named EGEDERS™ | present a good practice example aligned with ‘The Graduating European Dentist’ competencies |
| Yuka et al.(19) | / | 3 hours per times for 9 times in 4 weeks | 3 hours per times for 9 times in 4 weeks | Both groups studied in group | Zoom® combined with an LMS (WebClass®), lecture, videos | Improve the level of knowledge acquisition on removable prosthodontics |
| Li et al.(50) | / | 6 months | 6 months | None | Textbook | Learn about oral and maxillofacial oncology; Train students’ comprehensive abilities |
